# LncRNA weighted gene co-expression network analysis reveals novel biomarkers related to prostate cancer metastasis

**DOI:** 10.1186/s12920-022-01410-w

**Published:** 2022-12-13

**Authors:** Miao Liu, Man-Yun Chen, Jia-Meng Huang, Qian Liu, Lin Wang, Rong Liu, Nian Yang, Wei-Hua Huang, Wei Zhang

**Affiliations:** 1 Department of Clinical Pharmacology, Xiangya Hospital, Central South University, 87 Xiangya Road, 410008 Changsha, People’s Republic of China; 2 Institute of Clinical Pharmacology, Hunan Key Laboratory of Pharmacogenetics, Central South University, 110 Xiangya Road, 410078 Changsha, People’s Republic of China; 3 National Clinical Research Center for Geriatric Disorders, 87 Xiangya Road, Hunan 410008 Changsha, People’s Republic of China

**Keywords:** lncRNA, Prostate cancer, Metastasis, WGCNA, Systems biology

## Abstract

**Background:**

Most prostate cancer patients die from metastasis and lack accurate efficacious biomarkers to monitor the disease behavior, optimize treatment and assess prognosis. Herein, we aimed to identify meaningful lncRNA biomarkers associated with prostate cancer metastatic progression.

**Methods:**

By repurposing microarray probes, 11,624 lncRNAs in prostate cancer were obtained from Gene Expression Omnibus  database (GSE46691, *N* = 545; GSE29079, *N* = 235; GSE94767, *N* = 130). Weighted gene co-expression network analysis was applied to determine the co-expression lncRNA network pertinent to metastasis. Hub lncRNAs were screened. RNA-seq and clinical data from the Cancer Genome Atlas prostate cancer (TCGA-PRAD) cohort (*N* = 531) were analyzed. Transwell assay and bioinformatic analysis were performed for mechanism research.

**Results:**

The high expression levels of nine hub lncRNAs (FTX, AC005261.1, NORAD, LINC01578, AC004542.2, ZFAS1, EBLN3P, THUMPD3-AS1, GAS5) were significantly associated with Gleason score and increased probability of metastatic progression. Among these lncRNAs, ZFAS1 had the consistent trends of expression in all of the analysis from different cohorts, and the Kaplan-Meier survival analyses showed higher expression of ZFAS1 was associated with shorter relapse free survival. In-vitro studies confirmed that downregulation of ZFAS1 decreased prostate cancer cell migration.

**Conclusion:**

We offered some new insights into discovering lncRNA markers correlated with metastatic progression of prostate cancer using the WGCNA. Some may serve as potential prognostic biomarkers and therapeutic targets for advanced metastatic prostate cancer.

**Supplementary Information:**

The online version contains supplementary material available at 10.1186/s12920-022-01410-w.

## Introduction

According to the Encyclopedia of DNA Elements (ENCODE) project, less than 2% of the human genome is translated into proteins. More than 80% is transcribed into a versatile group of RNA. Those RNA transcripts without protein-coding capability, known as non-coding RNA (ncRNA), can be divided into “housekeeping ncRNA” such as mRNA and “regulatory ncRNA” such as small ncRNAs or long ncRNAs (lncRNAs) [1]. However, the functional roles of lncRNAs (> 200 nucleotides) are still not nearly as well-known as small ncRNAs, particularly miRNA. It gradually becomes clear that lncRNAs play important roles in transcription, translation, splicing, nuclear/cytoplasmic trafficking and interact with major pathways controlling proliferation, differentiation, or survival [2]. Accumulating evidence in hematological and solid malignancies have shown that lncRNAs can regulate tumor initiation, progression, or metastasis due to alterations of gene expressions [3]. In addition, for their tissue and tumor specificity, lncRNAs can be used as promising candidates for biomarkers and therapeutic targets.

Some lncRNAs have been applied successfully in clinical practice. Prostate cancer antigen 3 (PCA3), the highly prostate-specific lncRNA overexpressed in more than 95% of primary prostate tumors, was developed as a urine test for the supplementary diagnosis of prostate cancer (PCa) [4]. Furthermore, the Progensa PCA3 test was successfully approved by the US Food and Drug Administration (FDA) in 2012 [5]. The lncRNA Second Chromosome Locus Associated with Prostate 1 (SChLAP1) has also been identified as a molecular driver and predictive biomarker of aggressive PCa in multiple clinical studies [6]. PCa is the foremost non-cutaneous malignancy in men worldwide, with increasing morbidity and mortality. The leading cause of prostate cancer death is metastasis. The five-year survival rate for metastatic prostate cancer (mPCa) dramatically decrease to only 31% while for localized PCa is almost 100%^1^. Worse, some of them exhibit rapid resistance to standard androgen deprivation therapy (ADT) combined with/without microtubule-targeted taxane chemotherapy and lead to fatal disease. It is important to study clonal diversity and cancer heterogeneity alongside the development and impact of the tumour microenvironment (TME), which includes the extracellular matrix (ECM),blood vessels, immune cells and fibroblasts^2^. However, the present clinical predictors (such as serum prostate-specific antigen, biopsy Gleason score) of biological outcomes for PCa still remain imprecise and imperfect [7]. Meanwhile, molecular biomarkers, especially non-coding genes, set a new phenotypic frontier in prostate cancer metastasis and resistance^3^. Therefore, we aimed to find the clinically relevant lncRNA network and hub lncRNAs tightly associated with PCa metastasis by applying systems biology.

The functions of lncRNAs are greatly influenced by their transcript abundance due to not encoding proteins. However, the overall expression abundance of lncRNA is lower than mRNA, which leads to higher requirements for the amount of sequencing data for the detection of lncRNA. In comparison, chips have higher reliability in detecting low-abundance RNA, particularly in lncRNA with overlapping regions of mRNA. Chip-based probes are shorter and can be designed according to specific sequences, which are more suitable for high-throughput lncRNA detection than RNA sequencing. Therefore, we developed a computational pipeline to re-annotate probes that are uniquely mapped to lncRNA.

Moreover, the Affymetrix Human Exon 1.0 ST Array platform was used to query the expression of lncRNA in PCa samples. This platform retains the most annotated lncRNA data currently [8]. Based on the expression data, weighted gene co-expression network analysis (WGCNA), which allows a systems-level investigation of correlations between genotype and phenotype, was chosen to examine how lncRNAs jointly affect prostate cancer metastasis. WGCNA approach can identify the highly correlated genes that share quite similar expression profiles on different samples and construct a “co-expression network” using these genes. WGCNA can cluster highly correlated genes into a module, a subregion of the network, and might be linked to specific functions for the members involved in well-defined pathways. The central genes in a module are defined as “hub genes” that are regarded as the most suitable candidates to imply some physiological or pathological processes [9, 10]. Comparing co-expression lncRNAs networks according to the metastatic and non-metastatic associated expression data can recognize the distinguished modules and the most relevant hub lncRNAs. Thus, some potential diagnostic or prognostic lncRNAs that are highly associated with PCa metastasis could be found.

## Materials and methods

### Data recruitment and preprocessing

PCa related gene expression data were searched in the Gene Expression Omnibus (GEO, http://www.ncbi.nlm.nih.gov/geo/) of National Center of Biotechnology Information (NCBI), and were downloaded according to the accession numbers GSE46691(*N* = 545), GSE29079 (N = 235), GSE94767 (*N* = 130). The microarray platform used in these above datasets was Affymetrix Human Exon 1.0 ST array (HuEx-1_0-st). GSE46691 consisting of 545 PCa patients, 212 (39%) developed metastatic progression after long-term follow-up (mean 13.4 years), was used to construct the co-expression gene module and discover hub lncRNAs. GSE29079 and GSE94767 consisting of the tumor stage information, were exploited as independent validation datasets. These datasets were all labeled with their corresponding accession numbers. For the TCGA prostate adenocarcinoma (PRAD) cohort (*N* = 531), the gene expression profiles of the prostate cancer tumor and adjacent normal tissue and the most recent clinicopathological information of these patients were extracted from Genomic Data Commons Data Portal (https://portal.gdc.cancer.gov).

### Re-purposing data to interrogate lncRNA expression

We collected lncRNA annotations from the Ensembl database (Homo sapiens GRCh38, release 96) [11] and re-annotated probe sets of the Human Exon 1.0 ST array (GPL5188 or GPL5175) for lncRNAs through mapping all probes to the human genome with SeqMap [12], just as the computational pipeline developed by Liu et al. [8]. Only the probes with no mismatch and mapped uniquely to the genome were kept. Based on the Ensembl database annotations (http://www.ensembl.org/), probes mapped to pseudogene transcripts or protein-coding transcripts were all removed. At last, we obtained 11,624 corresponding lncRNA genes with no less than 4 probes. The raw intensity of the array probes was preprocessed identically using the R package oligo for normalization and background correction. The lncRNA expression level was measured as the average value of the background-corrected intensity with all probes corresponding to this lncRNA.

### WGCNA for lncRNA and hub lncRNAs definition

Four co-expression networks were constructed using the WGCNA package in R. LncRNA expression profiles of the GSE46691 dataset were used to conduct the co-expression networks of the selected lncRNAs. An unsupervised co-expression relationship was initially built on the adjacency matrix of connection strengths using Pearson’s correlation coefficients for all pair-wise lncRNAs. The parameter of the matrix emphasized the strong connections between lncRNAs, whereas it penalized weaker connections (*β* = 6). Then, the adjacency matrix has been transformed into a topological overlap matrix (TOM), which represented the connectivity of each lncRNA in the module. Hierarchical cluster analysis dendrogram was also performed to identify co-expression clusters with the gene profiler with a minimum gene number of 30.

The module eigengenes (MEs) were calculated following principal components analysis, representing the mean expression level of a module. The association between tumor metastasis (metastasis = 1; non-metastasis = 0) and MEs were tested with a logistic regression model. For single-lncRNA association analysis, each lncRNA expression was dichotomized around its median expression, and then logistic regression analysis was performed. Meanwhile, we test the association between the expression of each lncRNA and metastasis. Gene significance (GS) of each lncRNA, which represented the biological significance of the lncRNA for metastasis, was calculated as minus log 10 of the *p* values [13]. In our study, hub genes were selected by the following norm, top 10 genes with the largest k.in the module and GS larger than 1.3 (the *p*-value of the association test < 0.05). The significance of differences between groups was assessed by Student’s t-test or one-way ANOVA. P values of statistical significance are represented as ∗*p* < 0 05, ∗∗*p* < 0 01.

### Survival analyses

To appraise the prognostic values of the hub lncRNAs, we explored the relationship between hub lncRNAs and survival by using the ‘survival’ R package on the TCGA PRAD dataset. Biochemical recurrence-free survival (RFS) was used as the survival endpoints respectively. Patients were divided into the metastatic group and the non-metastatic group according to the TNM stages defined by the AJCC 8th edition system. Each lncRNA was dichotomized into high and low expression subgroups around its median value in each group. RFS was determined and compared using the Kaplan–Meier and the log-rank (Mantel-Cox) test.

### Cell lines and cell culture

The human prostate cancer cell lines PC-3 and LNCaP were purchased from the Cell Resource Center of Shanghai Institutes for Biological Sciences, Chinese Academy of Sciences (Shanghai, PR China). The cells were cultured according to the instructions from American Type Culture Collection (ATCC). PC-3 and LNCaP cells were cultured in F-12 K medium (Biological Industries) and RPMI-1640 medium (Biological Industries), respectively, supplemented with 10% (v/v) fetal bovine serum (Biological Industries), 100 units/mL penicillin, and 100 mg/L streptomycin (Invitrogen).

### Plasmid and RNA interference

The siRNA targeting ZFAS1 (si-ZFAS1) was obtained from Ribobio (Guangzhou, PR China), while as control, the mammalian non-targeting siRNA (si-NC) was used. Appropriate siRNA oligos for ZFAS1 were transfected into PC-3 or LNCaP cells by using Lipofectamine RNAiMAX (Invitrogen) according to the manufacturer’s protocol. Cells were harvested and examined for PCR analysis to ensure the efficiency of siRNA treatment after 24 h transfection or harvested for transwell assay after 48 h transfection. Detailed information about the sequences of siRNA could be found in Additional file 1: Table S1.

### RNA extraction and real-time PCR analysis

Real-time quantitive RT-PCR was carried out to confirm the inhibition of lncRNA expression. Briefly, total RNA was isolated with the TRIzol reagent (Invitrogen) and reverse transcribed using PrimeScript RT reagent Kit with gDNA Eraser (TaKaRa) according to the manufacturer’s instructions. Then the PCR reaction was conducted at 95 °C for 2 min followed by 40 cycles at 95 °C for 15 s and 61 °C for 20 s using the SYBR^®^ Premix Ex TaqTM II real-time PCR system (TaKaRa). All gene expression levels in different treatments were represented relative to their relevant control (Ct) and normalized to GAPDH gene levels (^ΔΔ^Ct). All assays were repeated at least three times. The final data were presented as mean ± SEM, and *p* < 0.05 was considered a statistical difference. The sequences of primers were listed in Additional file 1:  Table S2.

### Transwell assay

For transwell assay, 2 × 10^4^ cells of each group were re-suspended in 200ml serum-free medium and planted in the upper chamber (8.0 mm pore, BD, USA), and 600ml of appropriate medium with 10% FBS was added to the lower chamber. After incubating for 24 h at 37 ℃, the medium in the upper chamber was removed. The attached cells in the lower section were fixed with 4% ice-cold polymethanol for 15 min before stained with 0.1% crystal violet for 30 min. The cells that migrated to the reverse side of the upper chambers were photographed. The migration rate was calculated by counting the migration cells in five random fields. All assays were repeated at least three times. The final data were presented as mean ± SEM, and *p* < 0.05 was considered a statistical difference.

### LncRNA–miRNA–mRNA network prediction

For predicting potential lncRNA–target regulations, we used LncTarD (http://bio-bigdata.hrbmu.edu.cn/LncTarD/), a manually curated database that offered a comprehensive resource of experimentally supported lncRNA-target regulations and integrated pan-cancer transcriptome data from TCGA[14]. The experimental module from DIANA-LncBase v2 (www.microrna.gr/LncBase), which provided a broad compendium of experimentally supported microRNA-lncRNA interactions from high-throughput and low yield methodologies extracted from published literature and analyzed raw sequencing data[15], was used to predict lncRNA-microRNA interactions (validation type was Direct). MiRcode (http://www.mircode.org)[16], which provides putative miRNA target sites based on the complete GENCODE gene annotation, and ctcRbase (http://www.origin-gene.cn/database/ctcRbase/), a database collected expression data of circulating tumor cells/microemboli (CTCs/CTM) in all kinds of cancer types derived from RNA-seq data analyses [17], were used to predict miRNA–mRNA target relationships. Cytoscape_3.7.2 software was used to visualize networks.

## Results

### LncRNA modules association with PCa metastasis

**Fig. 1:**
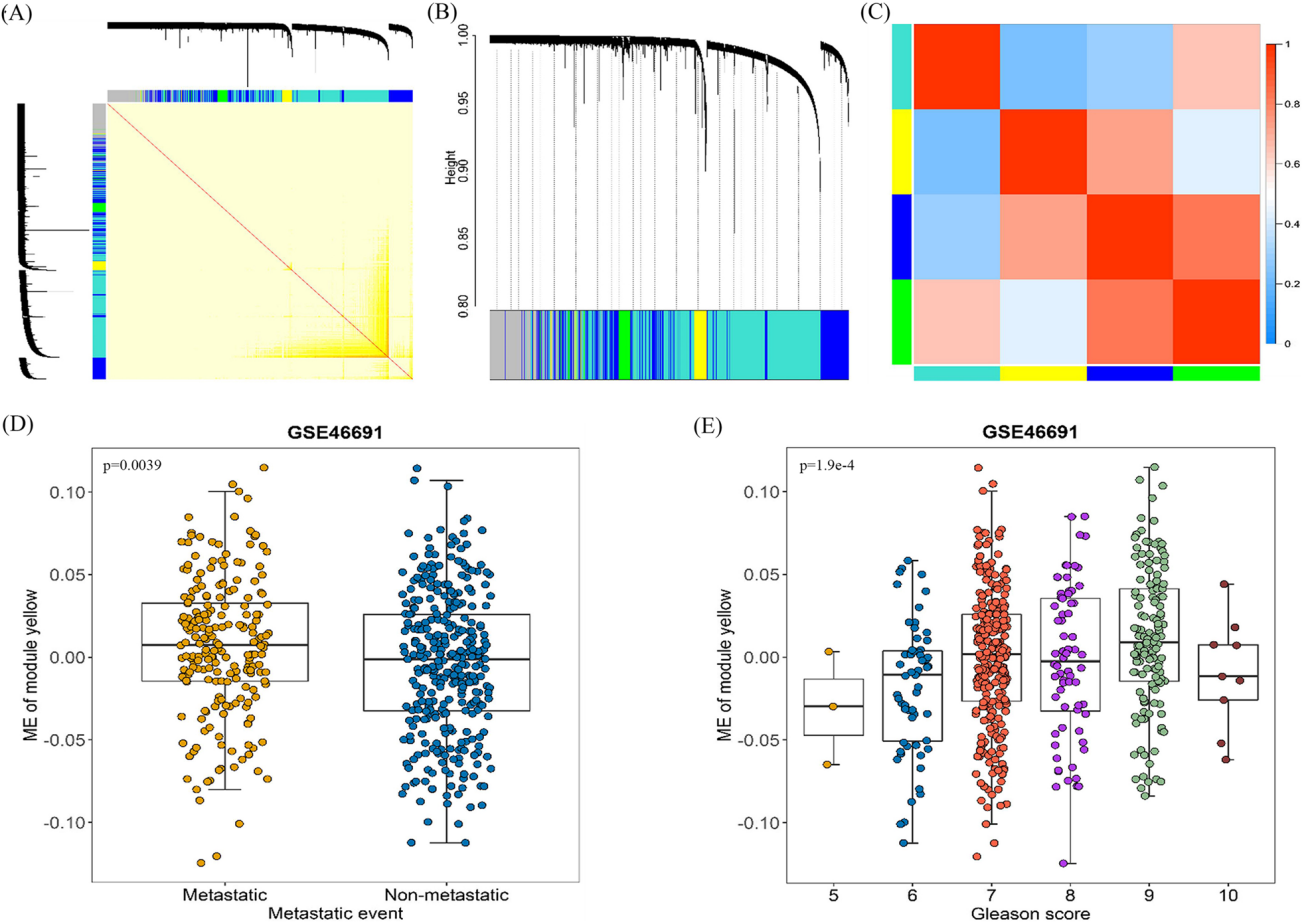
WGCNA of lncRNAs for PRAD. **A** Topological overlap matrix plot showing the lncRNA co-expression network (yellow part) and the modules based on co-expression network (left and up). On a linear scale, the depth of the yellow color and the intensity of the relevance between the pairs of modules have a positive correlation. Each column and row represent a lncRNA. **B** Clustering dendrograms of lncRNAs based on the topological overlap and the assigned module colors. Four co-expression modules were constructed with different colors, and the height reflects the affinity of individual lncRNAs. **C** Heatmap plot for the adjacencies in the eigengene network. Red represents positive correlation, and blue represents negative correlation. **D** ME of Module yellow-metastasis relationships. **E** ME of Module yellow- disease progression relationships

Totally, 4 lncRNA modules were identified, labeled with blue, green, turquoise, or yellow (Fig. 1 A, B), which indicated a high degree of independence among the 4 modules and relative independence of lncRNAs expression in each module. Similar results were shown by the heatmap plotted according to the correlation between the 4 modules (Fig. 1 C). The yellow module (OR = 1.44, 95% CI = 1.02–2.04, *p* = 3.83 × 10^− 2^) was significantly associated with PCa metastasis (Table 1). The MEs in the yellow module was calculated and revealed a positively correlation with tumor metastasis status (*p* = 0.0039) as well as the Gleason score(*p* = 1.9 × 10^− 4^) (Fig. 1D, E).

**Table 1:** Association between modules and metastasis in GSE46691

Module	Gene count	OR (95% CI)	*p*-value
blue	2868	1.20 (0.848–1.69)	0.31
green	483	0.994 (0.704–1.403)	0.97
turquoise	5939	0.78 (0.55–1.095)	0.15
yellow	541	1.44 (1.02–2.04)	0.038

### Hub lncRNAs definition and correlation with metastasis

**Fig. 2:**
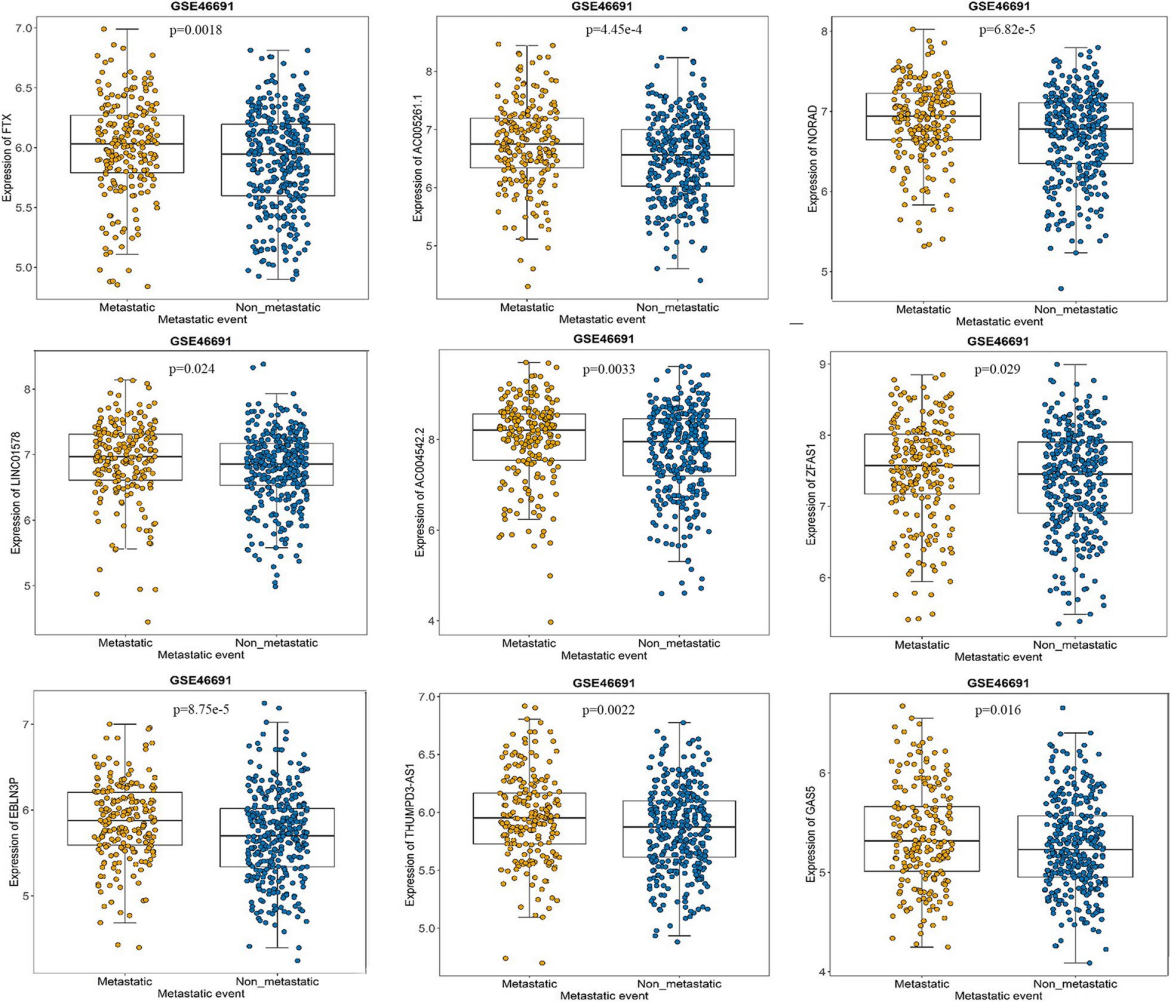
Differentially expressed hub lncRNAs in metastasis and non- metastasis tissues. Left: Log10 expression data in 212 metastasis PCa samples. Right: Log10 expression data in 333 non-metastasis PCa samples

9 lncRNAs (FTX, AC005261.1, NORAD, LINC01578, AC004542.2, ZFAS1, EBLN3P, THUMPD3-AS1, GAS5) in the yellow module met the criteria (Table 2). The Results indicated that the expression of all the 9 hub lncRNAs was up-regulated in metastatic tumor group relative to non-metastatic tumor group (Fig. 2).

**Table 2:** Association relationship between hub genes with metastasis in the training datasets

Ensemble id	Gene Symbol	Position	k.in rank	OR (95% CI)	*P* value
ENSG00000230590	FTX	chrX:73,940,435–74,293,574	1	1.99 (1.30–3.09)	1.82 × 10^− 3^
ENSG00000268205	AC005261.1	chr19:57304305–57,308,562	2	1.57 (1.22–2.03)	4.45 × 10^− 4^
ENSG00000260032	NORAD	chr20:36045618–36,051,018	3	2.05 (1.45–2.94)	6.82 × 10^− 5^
ENSG00000272888	LINC01578	chr15:92882707–92,899,701	4	1.43 (1.05–1.96)	2.42 × 10^− 2^
ENSG00000269987	AC004542.2	Chr22: 30,976,515–30,978,848	5	1.33 (1.10–1.62)	3.25 × 10^− 3^
ENSG00000177410	ZFAS1	chr20:49278178–49,295,738	6	1.32 (1.03–1.70)	2.90 × 10^− 2^
ENSG00000281649	EBLN3P	chr9:37079857–37,090,507	7	2.07 (1.45–3.00)	8.75 × 10^− 5^
ENSG00000206573	THUMPD3-AS1	chr3:9349689–9,398,579	8	2.11 (1.31–3.44)	2.22 × 10^− 3^
ENSG00000234741	GAS5	chr1: 173,858,559–173,868,882	9	1.57 (1.09–2.27)	1.61 × 10^− 2^

### Altered expression of hub lncRNAs throughout prostate tumor progression

**Fig. 3:**
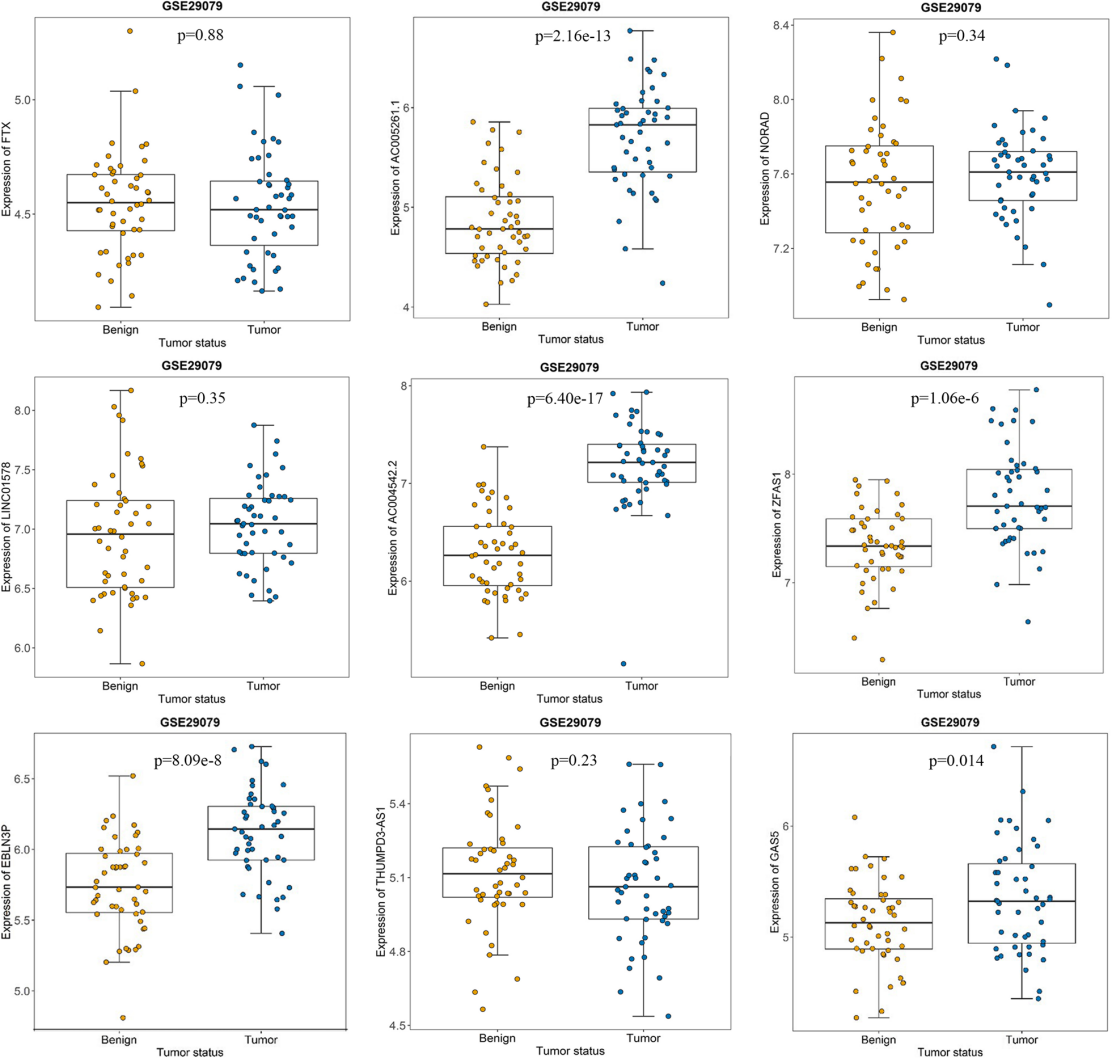
Differentially expressed hub lncRNAs in different tumor grades of PCa cohort (GSE29079). Expression levels of FTX, AC005261.1, NORAD, LINC01578, AC004542.2, ZFAS1, EBLN3P, THUMPD3-AS1, and GAS5 in benign and tumor tissues

**Fig. 4:**
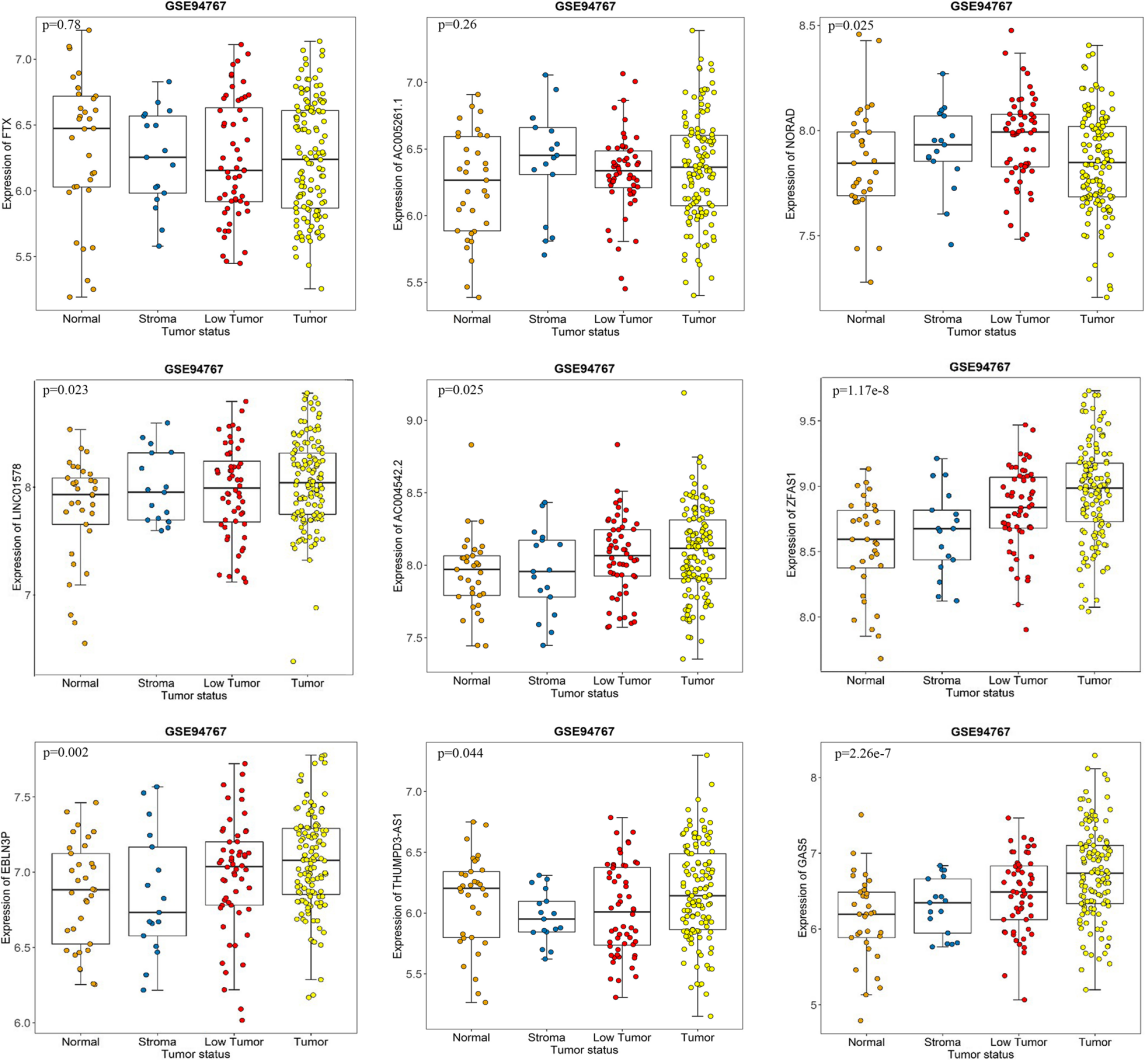
Differentially expressed hub lncRNAs in different tumor grades of PCa cohort (GSE94767). Expression levels of FTX, AC005261.1, NORAD, LINC01578, AC004542.2, ZFAS1, EBLN3P, THUMPD3-AS1, and GAS5 in normal, stroma, low tumor and tumor tissues

According to GSE29079 dataset, the samples were divided into the neoplasm and benign groups, *T*-test analysis showed that 5 lncRNAs (AC005261.1 *p* = 2.16 × 10^− 13^, AC004542.2 *p* = 6.40 × 10^− 17^, ZFAS1 *p* = 1.06 × 10^− 6^, EBLN3P *p* = 8.09 × 10^− 8^, GAS5 p = 0.014) had been up-regulated in the tumor samples, while other 4 lncRNAs were not significant (Fig. 3). Furthermore, the one-way ANOVA analysis showed seven lncRNAs increased with tumor progression (NORAD *p* = 0.025, LINC01578 *p* = 0.023, AC004542.2 *p* = 0.025, ZFAS1 *p* = 1.17 × 10^− 8^, EBLN3P *p* = 0.002, THUMPD3-AS1 *p* = 0.044, GAS5 *p* = 2.26 × 10^− 7^), which implied the expression levels of these lncRNAs had a highly consistent variation tendency with the grades of PCa (Fig. 4). Taken together, the lncRNAs in common (EBLN3P, AC004542.2, ZFAS1, and GAS5) seemed the most potentially predictive or prognostic genes for PCa that were closely related to the tumor progression.

### Hub lncRNAs validation using TCGA data

**Fig. 5:**
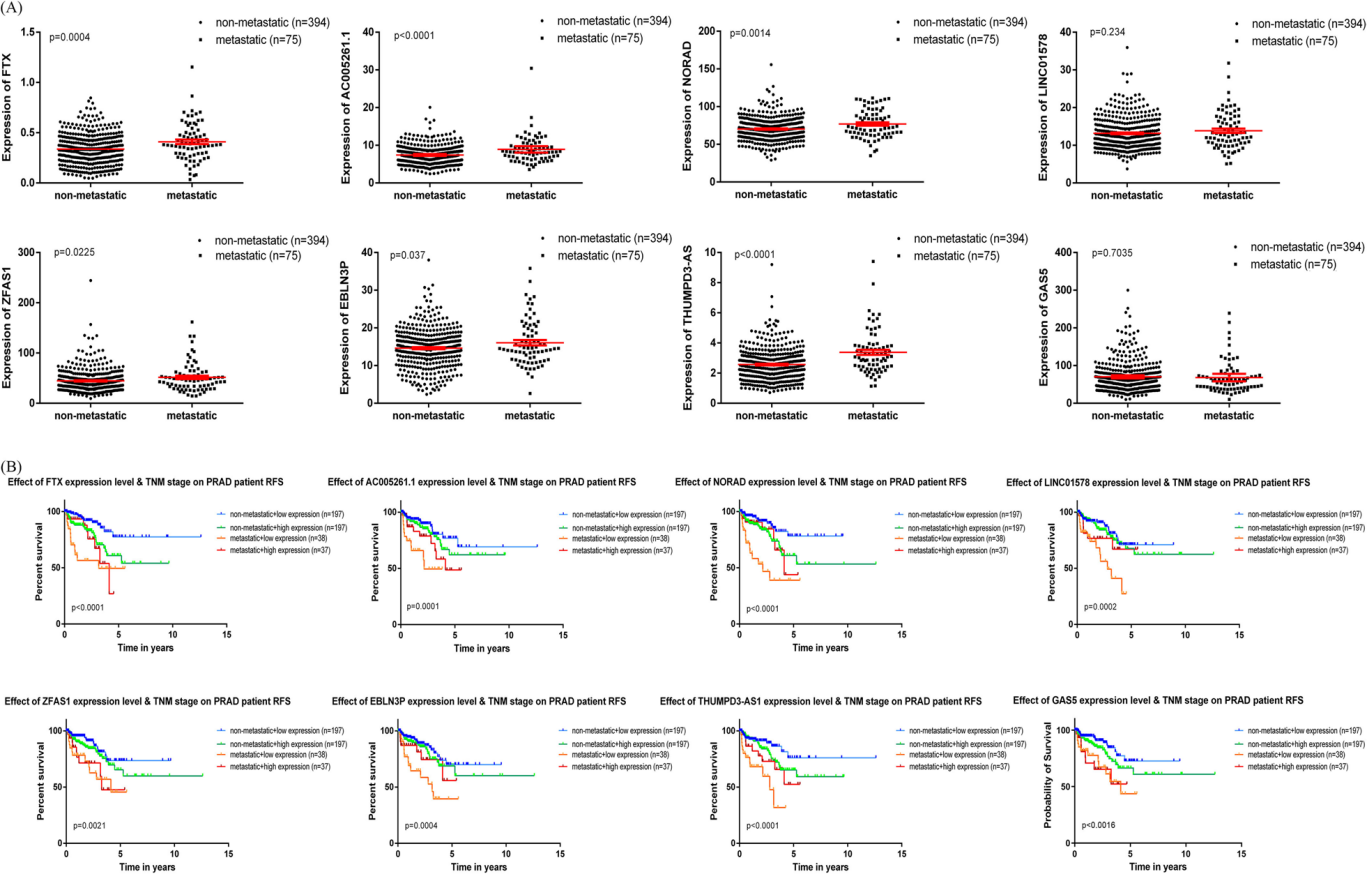
Hub lncRNAs validation in TCGA data. **A**The expression levels of FTX, AC005261.1, NORAD, LINC01578, ZFAS1, EBLN3P, THUMPD3-AS1, and GAS5 in non-metastatic and metastatic groups. **B** RFS curve of the 8 hub lncRNAs in PCa based on Kaplan–Meier analysis and log-rank test. Patients were divided into the high expression level group and the low expression level group based on the median value in non-metastatic or metastatic group

The results showed that AC005261.1, LINC01578, ZFAS1, EBLN3P, THUMPD3-AS1 and GAS5 were highly expressed in tumor than adjacent normal samples (Additional file 2:  Fig. S1). Differential expressions of FTX and NORAD were not significant. However, when the patients were stratified into metastatic and non-metastatic groups according to TNM classification (N > 0 or M > 0), the expressions of FTX, AC005261.1, NORAD, ZFAS1, EBLN3P and THUMPD3-AS1 were significantly higher in metastatic group (Fig. 5 A). Interestingly, when the patients were classified into two categories determined by tumor extra-prostatic extension or Gleason score > 8, Stage I-IIIA and Stage IIIB-IV, only LINC01578 and GAS5 were not significant higher expressions in high-grade tumors (Additional file 3:  Fig. 2 A).

The curves revealed that higher expression of almost each hub lncRNA in non-metastatic group was connected to shorter RFS. Conversely, lower expression of almost each of them in metastatic group was closely correlated with worse survival (Fig. 5B). But unexpectedly, the expressions of these hub lncRNAs except FTX seem unable to predict the RFS in advanced patients when the patients were classified into Stage I-IIIA and Stage IIIB-IV group (Additional file 3:  Fig. 2B). These results implied the 8 hub lncRNAs might be involved in the pathogenesis of tumor metastasis, while their functions were likely to change once the tumor had metastasized.

### Downregulation of ZFAS1 inhibited the migration of prostate cancer cells

**Fig. 6:**
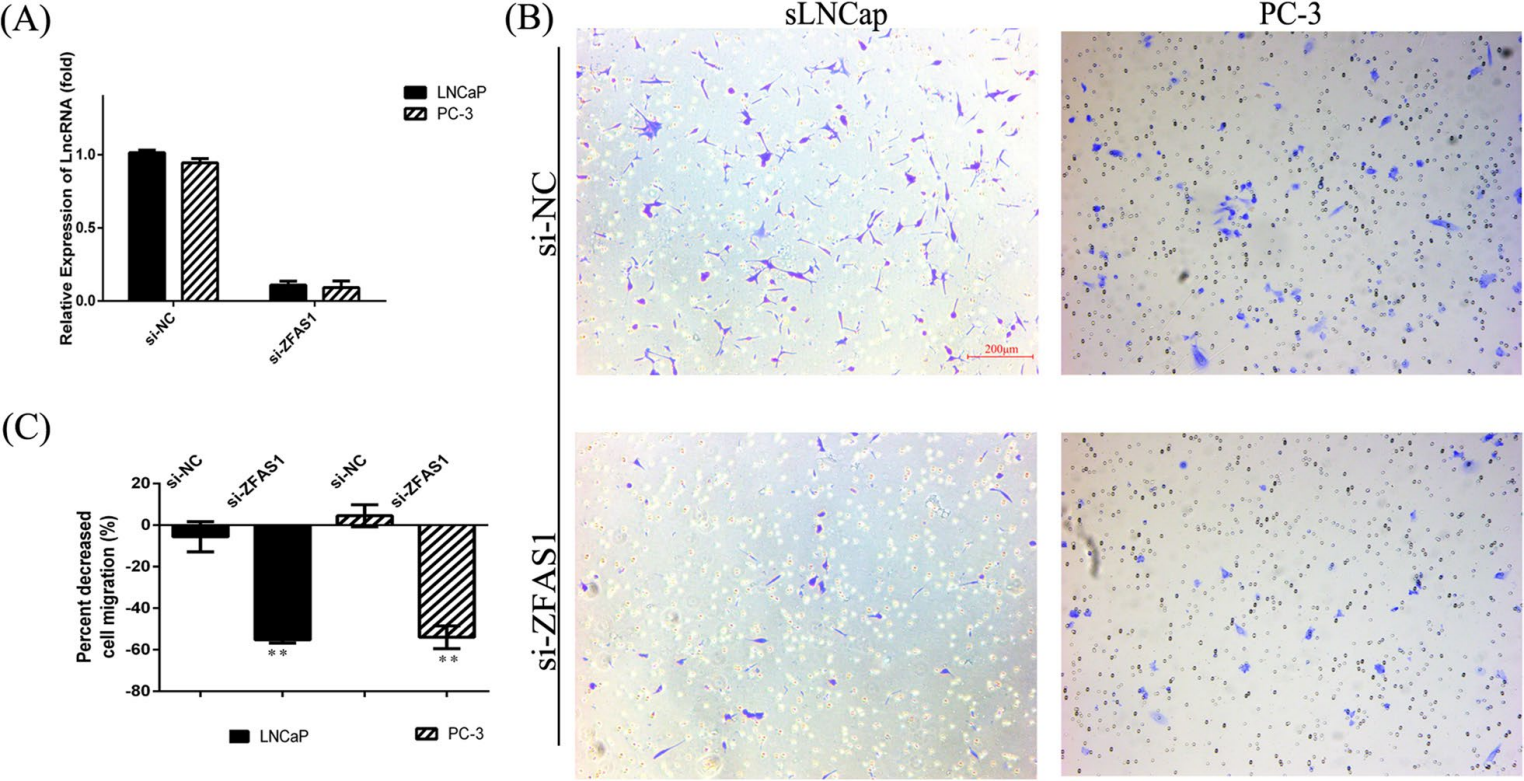
Downregulation of the expression of ZFAS1 diminishes cellular migration in vitro. **A** PCR analysis of expression levels of ZFAS1 in LNCaP or PC-3 cells treated with si-ZFAS1 compared with negative control cells **B** Representative of inhibitive effect of downregulation of ZFAS1 on the migration of cells in a transwell assay.**C** Qualitative analysis of transwell assay. Data presented were mean ± SEM of three independent experiments. * *p* < 0.05, ** *p* < 0.01

In view of above findings, the expression of ZFAS1 was consistent in all different independent cohorts. To further verify the its effect on prostate cancer metastasis, the mobility of cells was measured by the transwell experiment. Downregulation of ZFAS1 showed a decreased migratory capacity of prostate cancer cells (Fig. 6). Compared with negative control cells, there was a significant 55%, or 54% decrease in LNCaP cells or PC-3 cells transfected with si-ZFAS1, respectively. The changes in migratory cellular behavior impled that downregulation of ZFAS1 in prostate cancer might cause a less aggressive phenotype consistent with the clinical data analysis.

### lncRNA–miRNA–mRNA ceRNA predicted regulatory networks

**Fig. 7:**
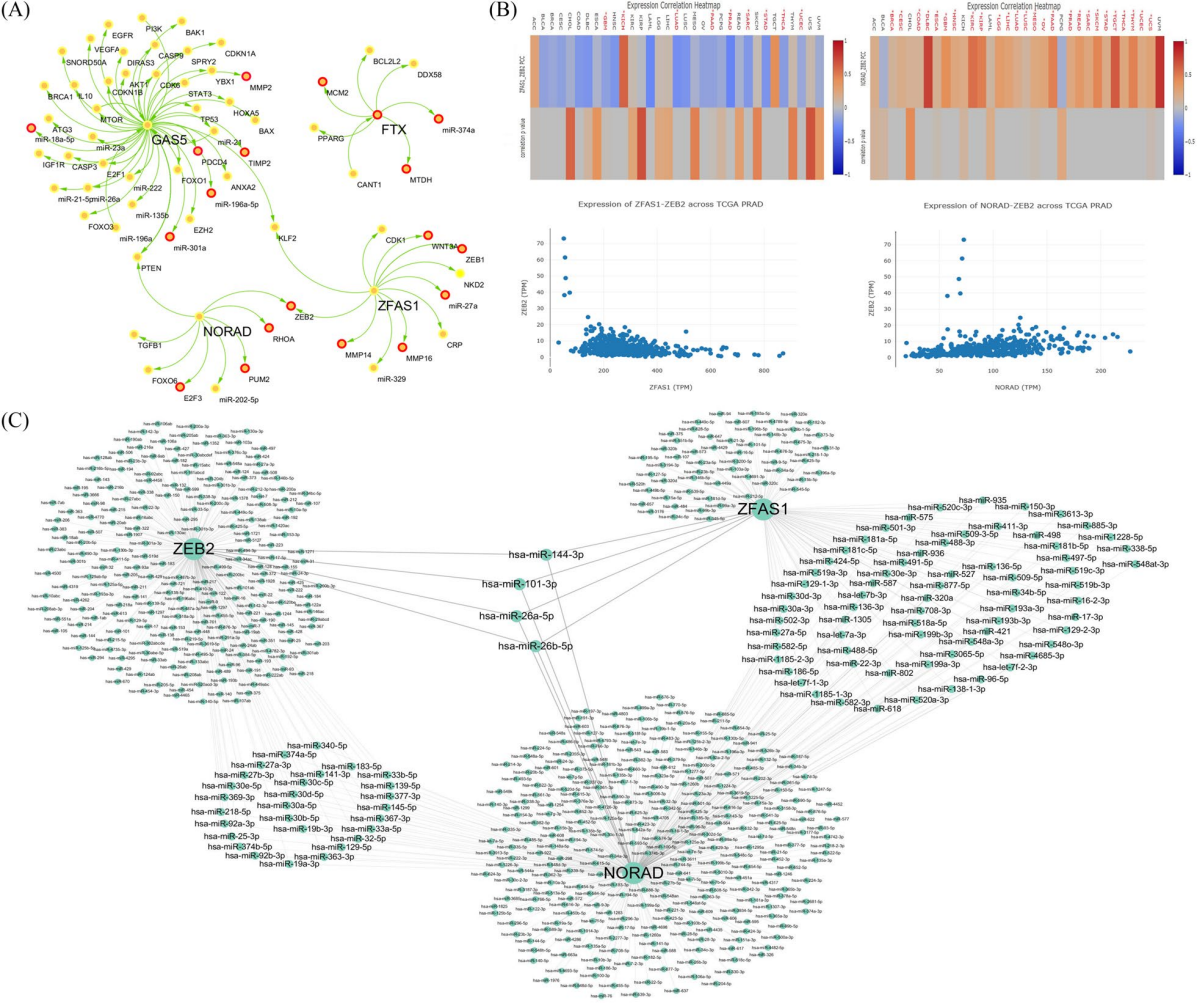
The lncRNA–miRNA–mRNA ceRNA predicted regulatory networks. **A** Network of hub lncRNA-target regulations. The red circle represents the target gene-related to cell metastasis based on experimental data. **B** Pearson correlation coefficients between ZFAS1 and ZEB2 or between NORAD and ZEB2 in each of 33 TCGA cancer types and scatter plot of ZFAS1-ZEB2 or NORAD-ZEB2 expression in PRAD cancer type, red*represents the significant association. **C** lncRNA–miRNA–mRNA networks base on the computationally/ experimentally supported predicted interacting miRNAs of ZFAS1, NORAD, and their common potential target ZEB2. The circle size depends on the EdgeCount

Based on the LncTarD records, we obtained the target networks of 4 hub lncRNAs (GAS, ZFAS1, NORAD, and FTX) (Fig. 7 A). It was noted that ZEB2 appeared to be not only co-regulated by NORAD and ZFAS1 but also involved in cell migration. The expression correlations of ZFAS1-ZEB2 and NORAD-ZEB2 across 33 TCGA cancer types were checked (Fig. 7B). Interestingly, ZFAS1 was negatively correlated with ZEB2, while NORAD was positively correlated with ZEB2 in PRAD cancer type. For mechanism research, the relevant lncRNA–miRNA–mRNA networks were constructed depending on the data from DIANA-LncBase, miRcode, and ctcRbase (Fig. 7 C). Among the potential miRNA targets, ZFAS1, NORAD, and ZEB2 (has-miR-144-3p, has-miR-101-3p, has-miR-26a-5p, has-miR-26b-5p) might be worthy of further verification.

## Discussion

LncRNAs have emerged as new important regulators in the pathogenesis of human urologic cancers over the last decade. Herein, we undertook an unbiased study to identify lncRNA biomarkers associated with prostate cancer metastatic progression. Microarray/RNA-seq and clinical data of prostate cancer were obtained from the GEO and TCGA-PRAD cohort. The WGCNA was used to identify lncRNA networks in GSE46691. One lncRNA module was confirmed to be associated with cancer metastasis, and nine hub lncRNAs, namely FTX, AC005261.1, NORAD, LINC01578, AC004542.2, ZFAS1, EBLN3P, THUMPD3-AS1, and GAS5, were discovered. These nine lncRNAs’ expressions were all up-regulated in metastasis samples and significantly associated with the Gleason score. Among them, ZFAS1was not only high expressed in tumor groups compared with adjacent normal/benign groups, but significantly increased with deterioration by verifying in all the cohorts. More potentially, survival analysis suggested that early PCa patients with high expression of ZFAS1 might have a worse prognosis. Taken together, ZFAS1 appeared to be involved in the development of tumor metastasis. The in vitro analysis further confirmed that down-regulation of ZFAS1 expression decreased prostate cancer cell migration. In addition, the hub lncRNA-target networks were constructed, which implied that ZFAS1 and NORAD might participate in the process of cell migration through their common target ZEB2.

Metastatic PCa remains a fatal condition when cancer cells spread to the pelvic, bladder, rectum, bone, retroperitoneal lymph nodes, spinal cord, and other body areas, despite localized PCa has a high long-term survival [18]. Metastatic PCa may fall into two broad classifications, disease treated with no androgen deprivation and disease resistant to such therapy. The standard of care for metastatic patients remains androgen-deprivation therapy, which uses gonadotropin-releasing hormone (GnRH) analogs as an alternative to surgical castration [19]. The diagnosis of the metastatic disease depends on imaging, including radionuclide bone scanning, computed tomography (CT), positron emission tomography (PET) with choline or prostate-specific membrane antigen (PSMA), whole-body magnetic resonance imaging (MRI). However, the pathological confirmation from imaging studies is incomplete and not sufficiently sensitive [20]. Second, While androgen deprivation therapy (ADT) combined with microtubule-targeted taxane chemotherapy can provide a survival benefit in recurrent or metastatic disease, treatment resistance invariably develops, leading to fatal disease. Acquired drug resistance is the result of genetic/epigenetic changes that confer a drug-resistant phenotype in cancer cells^4^. LncRNA mediated mechanisms have been associated with epigenetic changes in prostate cancer. According to the results of the COSMIC-021, combination of the cabozantinib, a kind of antiangiogenic drugs, with the immune checkpoint inhibitor atezolizumab achieved encouraging activity in patients with metastatic castration-resistant PC (mCRPC), while multiple previous phase III clinical trials^5,6^ in refractory CRPC have demonstrated increased toxicity with no clinical benefit. Tumor angiogenesis is regulated through interaction between anti-angiogenic factors and pro-angiogenic within the tumor microenvironment. A growing number of studies have shown that different lncRNAs affect tumor angiogenesis, for example, lncRNA H19 targets miR-199a-5p and negatively regulates VEGFA ^7^. The vast spectrum of cancer traits warrants better molecular biomarkers to identify the more aggressive and clinically significant tumor subtypes. Accumulating recent evidence has shown that lncRNAs play key roles in cancer initiation or progression and appear to have many features for diagnostic markers or therapeutic targets [5].

Similar to PCA3, the clinically-relevant lncRNAs in PCa continue to be discovered. Studies in two separate independent cohorts validated prostate cancer-associated transcript-14 (PCAT-14), an androgen-regulated lncRNA, might be used as a novel PCa diagnosis biomarker for its high expression prostate tumors and low expression linked to poor outcomes [21, 22]. Studies found that prostate cancer gene expression marker 1 (PCGEM1) with highly prostate-specificity was also related to androgen receptor (AR) signaling and was overexpressed in therapy-resistant PCa [23, 24]. Metastasis-associated lung adenocarcinoma transcript-1 (MALAT1) reported a close connection between high expression and poor prognosis indicators[25, 26]. Similar findings had been made with the second chromosome locus associated with prostate-1 (SCHLAP1) [27]. Other lncRNAs clinically relevant to PCa were such as urothelial carcinoma-associated 1 (UCA1) [28, 29], nuclear enriched abundant transcript 1 (NEAT1) [30], HLA complex group 11 (HCG11) [31]. Nevertheless, only a few lncRNAs related to PCa metastasis have been identified so far. Thus, we performed WGCNA by using publicly available data to characterize the metastasis-associated lncRNAs.

WGCNA, also known as the weighted correlation network analysis, is widely used in bioinformatics applications based on pairwise correlations amongst variables [9]. It has been extensively used in various genomic applications. WGCNA can also be used as a data reduction technique, clustering method, feature selection method, framework for integrating genomic data, and data exploratory technique [32]. According to the theory, genes involved in similar pathways or closely related functions may have similar expression profiles. The WGCNA approach as a systems biology strategy transforms gene expression data across samples into a co-expression module that can provide perception in signaling networks. The results from the GSE46691 dataset showed that nine hub lncRNAs had noticeable associations for metastasis in the yellow module by the WGCNA package tool. Though the original array design did not target lncRNA measurement, microarray probes could be re-annotated for interrogating the expression of lncRNA. Especially comparing with RNA-seq data, the array-based expression data had better detection sensitivity and lower technical variation due to the low abundance of lncRNA [32]. Expression analyses from the other two independent datasets (GSE29079, GSE94767) showed four (AC004542.2, ZFAS1, EBLN3P, GAS5) out of nine lncRNAs were significantly overexpressed in tumor. Moreover, the TCGA data also showed again that ZFAS1, EBLN3P, or GAS5 was overexpressed in PCa tumor compared to adjacent normal samples. Besides, ZFAS1 showed the potential to be a predictor of poor prognosis.

For further mechanism research, the hub lncRNA-target networks based on experimentally supported functional regulations and expression associations in human diseases from LncTarD were constructed. ZEB2 was found to be a common potential target for both ZFAS1 and NORAD. The expression of ZEB2 in the TCGA-PRAD dataset was negatively correlated with ZFAS1 and positively correlated with NORAD, which indicated that the “co-highly expressed” ZFAS1 and NORAD had more complicated molecular mechanisms for regulating cell metastasis. ZEB2, a key activator of epithelial-mesenchymal transition (EMT), has been found to promote EMT processes in various cancer metastases [33]. Moreover, NORAD and ZFAS1 have been reported to promote cell invasion and migration in cervical cancer and bladder cancer, respectively, by up-regulating ZEB2 [34, 35]. Detaching from the tumor mass and becoming motile are necessary for metastasizing cancer cells. Once these cells intravasate into the lymphatic or blood circulation, the survivors among them will attach to the target organ endothelium, extravasate into the organ parenchyma, and proliferate. The abnormal activation of an EMT contributes to cancer metastasis, so treatments targeting the EMT pathway have become an attractive strategy [36]. In order to investigate the regulatory mechanism among NORAD, ZFAS1 and ZEB2, DIANA-LncBase v2, miRcode, and ctcRbase databases were used to discover their respective potential target miRNAs. The results showed 69 common potential miRNA targets between ZFAS1 and NORAD and 28 common potential miRNA targets between NORAD and ZEB2. The four miRNAs of has-miR-144-3p, has-miR-101-3p, has-miR-26a-5p, and has-miR-26b-5p were potential targets shared by ZFAS1, NORAD, and ZEB2. There have been no reports about the involvement of these four miRNAs in tumor migration or invasion. As we know, multiple targets could be regulated by one miRNA, while one target could also be co-regulated by multiple miRNAs. Similarly, one lncRNA could target more than one miRNA, and some lncRNAs could form a complex lncRNA-miRNA-mRNA regulatory network [37]. In addition, the regulation of multiple lncRNAs on the same target gene may have differences in their response time.

Indeed, ZFAS1 was up-regulated in metastasis samples and related to poor RFS [38]. High expression of ZFAS1 was closely associated with worse disease-free survival for PCa or gliomas patients [39, 40]. Recent studies disclosed that miR-135a-5 and miR-150-5p could be used as binding targets of ZFAS1 to participate in the regulation of tumor proliferation, invasion, and migration [41, 42]. As an important mechanism of lncRNA function, some lncRNAs with miRNA-binding sites can serve as ‘sponges’ to seclude endogenous miRNAs, which disturbs the modulation of gene expression mediated by miRNA. This phenomenon called competing endogenous RNAs (ceRNAs) belongs to the genome-wide finetuning regulatory molecules network [43–45]. PTEN pseudogene (PTENP1) is one of the ceRNAs correlated with PCa, which is linked to PTEN phosphatase as a tumor-suppressive pseudogene. Because of the 3’ UTR of PTENP1 RNA binding the same region of regulatory miRNA sequences, PTENP1 could reduce the downregulation of PTEN mRNA [46]. At present, the research on the mechanism of ZFAS1 as ceRNA in migration is still in progress.

NORAD, a newly identified lncRNA, is unique for its high conservatism and the key role in maintaining chromosomal stability by modulating the Pumilio (PUM) proteins’ activity. Moreover, genome instability drives metastasis via a cytosolic DNA response [47]. Recent studies indicated that NORAD was highly expressed in numerous human cancers, including breast cancer, esophageal squamous cell carcinoma, pancreatic cancer, colorectal cancer, bladder cancer, and cervical cancer, with poor overall survival [34, 48–50]. However, in hepatocellular carcinoma, NORAD acts contradictorily as a tumor suppressor [51]. In the present research, our results suggested that NORAD might involve cell migration and be highly expressed in the metastasis community of PCa. Significantly, regulation of NORAD affects the PUM proteins and changes the extracellular vesicle (EV) proteins that participate in communication between cells in the tumor microenvironment [52, 53]. The effects of NORAD are implicated in almost all aspects of tumors, including carcinogenesis proliferation, apoptosis, invasion, and metastasis [54]. However, the precise molecular mechanisms of NORAD are still at a preliminary stage that requires further systematic investigation.

In summary, our analyses focused on the metastasis of PCa and uncovered some relevant hub lncRNAs. Although other lncRNAs did not show significant differences in all cohort like ZFAS1 that might be related to the different stages of the disease and the different types of samples we used for analysis. The other 8 hub lncRNAs still shown some potential as a biomarker correlated with metastatic progression of PCa. For ZFAS1, further molecular mechanism investigations are needed in order to understand its function adequately.

## Conclusion

To conclude, the yellow module derived from the microarray-based dataset via WGCNA was significantly associated with metastasis of PCa. The nine hub lncRNAs within yellow were successfully validated in different clinical cohots, especially ZFAS1. These results suggest some new lncRNAs as potential prognostic biomarkers and therapeutic targets for prostate cancer development and progression worthy of further investigation.

## Supplementary Information

**Additional file 1: Table S1. **siRNA and shRNA sequences. **Table S2.** Primer sequences of primers used in PCR analysis

**Additional file: 2 Fig. S1** Hub lncRNAs validation in TCGA-PRAD data. The expression levels of the 8 hub lncRNAs in adjacent normal and tumor tissues of PCa.

**Additional file : 3 Fig. S2** Hub lncRNAs validation in tumor tissues from TCGA-PRAD data AThe expression levels of the 8 hub lncRNAs in I-IIIA stage and IIIB-IV stage group of PCa. B RFS curve of the 8 hub lncRNAs in PCa based on Kaplan–Meier analysis and log-rank test. Patients were divided into the high expression level and the low expression level based on the median value in I-IIIA stage or IIIB-IV stage group.

## Data Availability

Publicly available datasets were analyzed in this study. All datasets for this study are included in the article. The authors acknowledge the efforts of all of the researchers who have contributed the data to the public databases of GEO (https://www.ncbi.nlm.nih.gov/geo/) with accession numbers GSE46691; GSE29079 and GSE94767, and the cancer genome database TCGA (https://www.cancer.gov/about-nci/organization/ccg/research/structural-genomics/tcga). The interpretation and reporting of these data are the sole responsibility of the authors.
