# Supplementary material for: LncRNA weighted gene co-expression network analysis reveals novel biomarkers related to prostate cancer metastasis
Source: BMC Med Genomics. 2022 Dec 13;15:256. doi: 10.1186/s12920-022-01410-w (PMC9745985; doi:10.1186/s12920-022-01410-w)
Supplement: Supplementary file 1 — Additional file 1: Table S1. siRNA and shRNA sequences. Table S2. Primer sequences of primers used in PCR analysis [file 12920_2022_1410_MOESM1_ESM.docx]

**Supplemental Tables**

**Supplemental Table S1. siRNA and shRNA sequences**

| **Gene** | **ID** | **Sequence** |
| --- | --- | --- |
| ZFAS1 | siRNA-5 | GCGTTTCGGGTCCAGTGCG |
| ZFAS1 | siRNA-6 | AGAAACTGGCGATGGAATA |
| ZFAS1 | siRNA-7 | GGATTTTGGAAGAGGGAGT |

**Supplemental Table S2. Primer sequences of primers used in PCR analysis**

| **Gene** | **Sequence (5'-3')** |
| --- | --- |
| ZFAS1 | Forward: AAGCCACGTGCAGACATCTA  Reverse: CTACTTCCAACACCCGCATT |
| GAPDH | Forward: GGAGCGAGATCCCTCCAAAAT  Reverse: GGCTGTTGTCATACTTCTCATGG |
