# Supplementary material for: LncRNA weighted gene co-expression network analysis reveals novel biomarkers related to prostate cancer metastasis
Source: BMC Med Genomics. 2022 Dec 13;15:256. doi: 10.1186/s12920-022-01410-w (PMC9745985; doi:10.1186/s12920-022-01410-w)
Supplement: Supplementary file 2 — Additional file: 2 Fig. S1 Hub lncRNAs validation in TCGA-PRAD data. The expression levels of the 8 hub lncRNAs in adjacent normal and tumor tissues of PCa. [file 12920_2022_1410_MOESM2_ESM.pptx]

## Slide 1
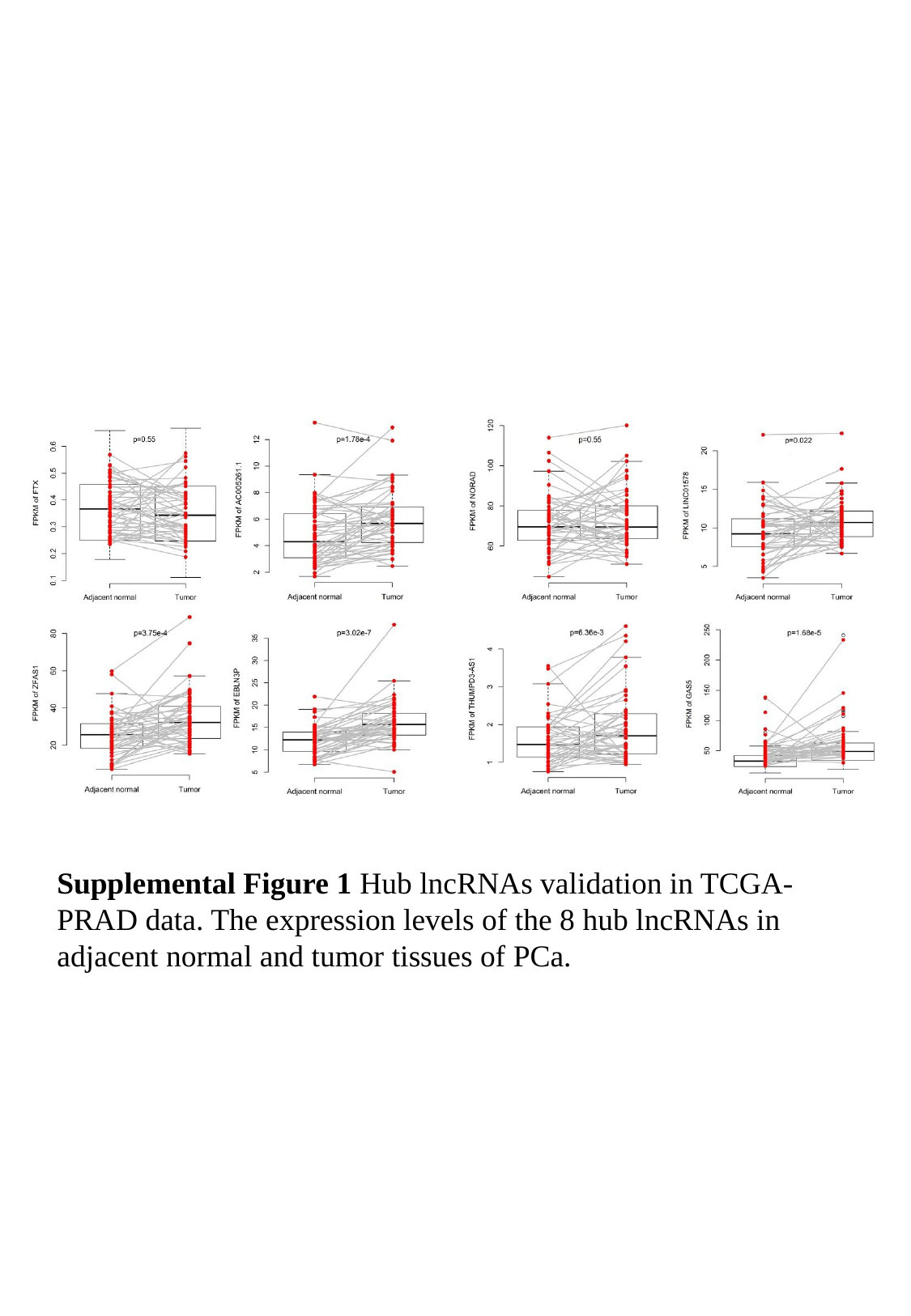

Supplemental Figure 1 Hub lncRNAs validation in TCGA-PRAD data. The expression levels of the 8 hub lncRNAs in adjacent normal and tumor tissues of PCa.
