# Supplementary material for: LncRNA weighted gene co-expression network analysis reveals novel biomarkers related to prostate cancer metastasis
Source: BMC Med Genomics. 2022 Dec 13;15:256. doi: 10.1186/s12920-022-01410-w (PMC9745985; doi:10.1186/s12920-022-01410-w)
Supplement: Supplementary file 3 — Additional file : 3 Fig. S2 Hub lncRNAs validation in tumor tissues from TCGA-PRAD data AThe expression levels of the 8 hub lncRNAs in I-IIIA stage and IIIB-IV stage group of PCa. B RFS curve of the 8 hub lncRNAs in PCa based on Kaplan–Meier analysis and log-rank test. Patients were divided into the high expression level and the low expression level based on the median value in I-IIIA stage or IIIB-IV stage group. [file 12920_2022_1410_MOESM3_ESM.pptx]

## Slide 1
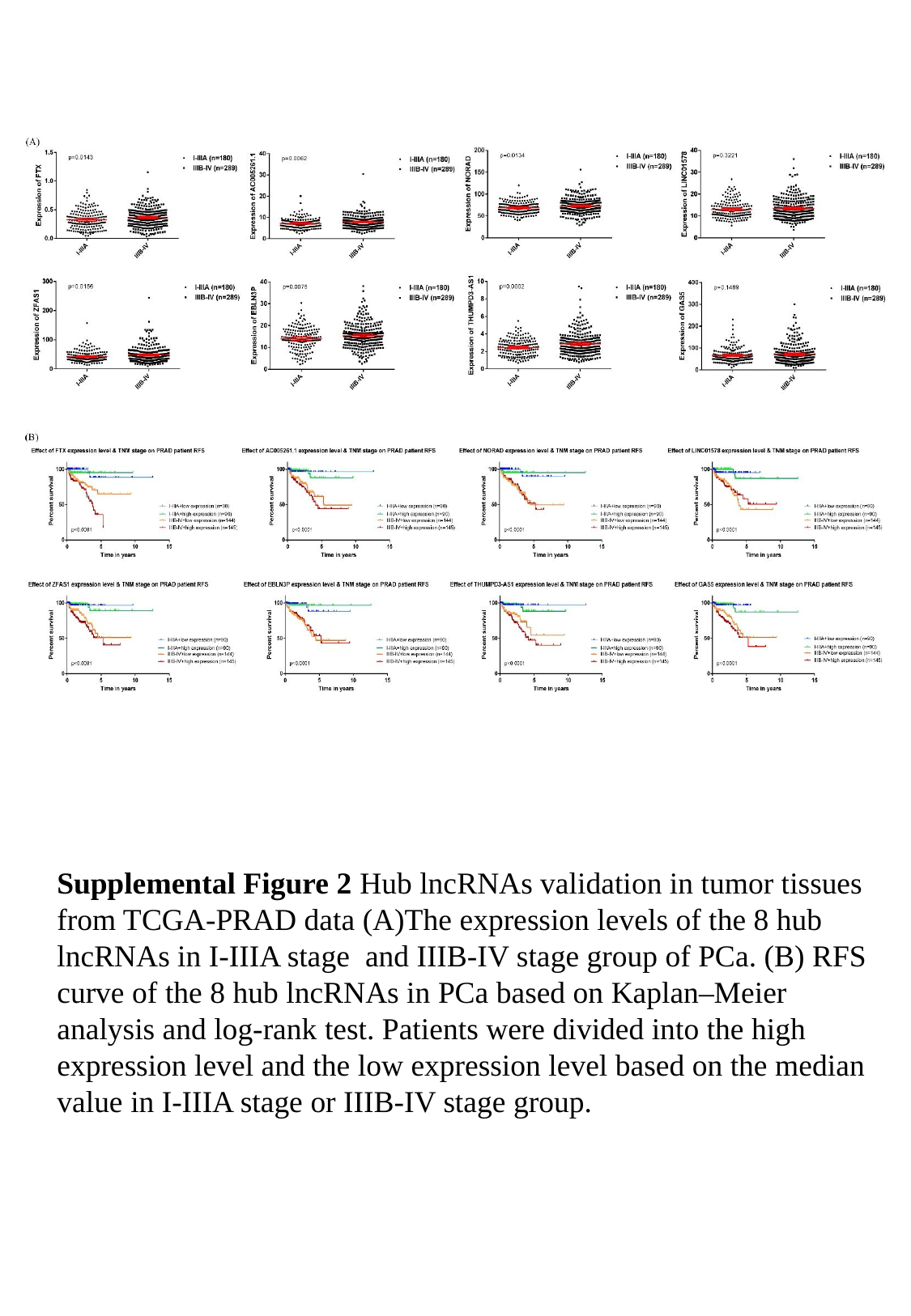

Supplemental Figure 2 Hub lncRNAs validation in tumor tissues from TCGA-PRAD data (A)The expression levels of the 8 hub lncRNAs in I-IIIA stage and IIIB-IV stage group of PCa. (B) RFS curve of the 8 hub lncRNAs in PCa based on Kaplan–Meier analysis and log-rank test. Patients were divided into the high expression level and the low expression level based on the median value in I-IIIA stage or IIIB-IV stage group.
